# The impact of school-based mitigation measures on the transmission of respiratory pathogens: the case of SARS-CoV-2

**DOI:** 10.1093/ije/dyag103

**Published:** 2026-07-09

**Authors:** Laura Fumanelli, Giorgio Guzzetta, Martina del Manso, Antonino Bella, Massimo Vicentini, Olivera Djuric, Eufemia Bisaccia, Pamela Mancuso, Silvia Cilloni, Elisabetta Larosa, Patrizio Pezzotti, Paolo Giorgi Rossi, Stefano Merler

**Affiliations:** Center for Health Emergencies, Bruno Kessler Foundation, Trento, Italy; DONDENA Centre for Research on Social Dynamics and Public Policy, Bocconi University, Milan, Italy; Center for Health Emergencies, Bruno Kessler Foundation, Trento, Italy; Department of Infectious Diseases, Istituto Superiore di Sanità, Rome, Italy; Department of Infectious Diseases, Istituto Superiore di Sanità, Rome, Italy; Epidemiology Unit, Azienda Unità Sanitaria Locale—IRCCS di Reggio Emilia, Reggio Emilia, Italy; Epidemiology Unit, Azienda Unità Sanitaria Locale—IRCCS di Reggio Emilia, Reggio Emilia, Italy; Centre for Biostatistics, Epidemiology, and Public Health (C-BEPH), Department of Clinical and Biological Sciences, University of Turin, Turin, Italy; Public Health Unit, Azienda Unità Sanitaria Locale—IRCCS di Reggio Emilia, Reggio Emilia, Italy; Epidemiology Unit, Azienda Unità Sanitaria Locale—IRCCS di Reggio Emilia, Reggio Emilia, Italy; Public Health Unit, Azienda Unità Sanitaria Locale—IRCCS di Reggio Emilia, Reggio Emilia, Italy; Public Health Unit, Azienda Unità Sanitaria Locale—IRCCS di Reggio Emilia, Reggio Emilia, Italy; Department of Infectious Diseases, Istituto Superiore di Sanità, Rome, Italy; Epidemiology Unit, Azienda Unità Sanitaria Locale—IRCCS di Reggio Emilia, Reggio Emilia, Italy; Center for Health Emergencies, Bruno Kessler Foundation, Trento, Italy

**Keywords:** schools, mitigation measures, impact assessment, respiratory infections, SARS-CoV-2, pandemic preparedness and response

## Abstract

**Background:**

School-based mitigation measures can curb transmission of severe respiratory pathogens but may also impose high societal costs. Understanding their impact at the population level is key for pandemic preparedness evaluations.

**Methods:**

We developed an individual-based model of SARS-CoV-2 transmission in households, schools, and the general community, calibrated to epidemiological estimates from complete contact tracing data collected in the province of Reggio Emilia, Italy, during March–April 2021 (a period characterized by the dominance of the Alpha variant and strict community restrictions). We quantified the impact of school-based mitigation measures, including distance-learning mandates, reactive class quarantines, and periodic screening.

**Results:**

Unmitigated transmission in schools would increase the population-level SARS-CoV-2 reproduction number from 0.94 (95% confidence interval [CI]: 0.84–1.03) to 1.38 (95% CI: 1.29–1.47), substantially amplifying COVID-19 morbidity and mortality. Intensive contact tracing with reactive class quarantines would reduce disease burden but would be insufficient to prevent widespread epidemic growth. Weekly universal screening of students and teachers with highly specific rapid tests, on top of contact tracing and reactive class quarantines, could effectively reduce the spread of infections and maintain a high level of in-person education.

**Conclusions:**

For highly transmissible severe respiratory infection with significant cryptic transmission, distance learning mandates may be necessary to protect vulnerable populations. However, weekly screening in schools may offer a viable strategy to preserve in-person education.

Key Messages

- We sought to assess the population-level impact of school-based mitigation measures against respiratory infections, aiming to identify strategies that could maximize in-person school attendance while limiting overall disease burden.
- During respiratory epidemics of public-health concern, widespread transmission within schools will eventually affect the broader population, including its most vulnerable groups, even under strict community measures; however, periodic screening of students and school staff, combined with reactive class quarantines, can enable epidemic control if rapid diagnostic tests are sufficiently accurate.
- When population immunity is low, distance-learning mandates may be required to curb the spread of a highly transmissible respiratory pathogen; investing in proactive, systematic interventions such as periodic mass screening in schools may contain the spread of disease while avoiding the substantial social costs of disrupting in-person education.

## Introduction

The transmission of respiratory infections in schools is facilitated by the high number of social contacts among students [1] and by their prolonged co-location in closed and often poorly ventilated classrooms [2]. Infections transmitted in schools among children and adolescents can lead to subsequent onward spread to the rest of the population via transmission in households and in the general community. The full extent of the contribution of schools to the overall disease burden must be quantified to evaluate the effectiveness of control and mitigation measures in these settings. For pandemic influenza, the critical importance of school-based mitigation measures has been clearly demonstrated [3–5]. Schools were therefore a natural focus of early interventions in the wake of the SARS-CoV-2 emergence in many countries, especially considering that pandemic preparedness plans had largely been based on influenza [6]. Nearly 1.3 billion learners worldwide (82% of the total enrolled) were not receiving in-person education in mid-April 2020, and 130 million (8.3%) 1 year later [7]. Extensive school closures entail significant social costs, including educational disruption, adverse mental health outcomes for children, economic and psychological challenges for adults responsible for childcare, and increased work absenteeism [8–10]. In the case of SARS-CoV-2, school closures have faced criticism because children and adolescents might have been less susceptible to infection [11, 12] and had remarkably lower rates of severe disease [13], thereby suggesting that the societal costs of suspending in-person education might not have been sufficiently balanced by epidemiological benefits.

Several studies have attempted to quantify the effect of SARS-CoV-2 school transmission [14, 15] and to evaluate the impact of school-based measures [16–29] by analyzing epidemiological data through the lens of mathematical models. These studies have focused on school-transmitted infections or on spillover to household contacts, while the effect of onward spread to the general community was not taken into account, except under hypothetical assumptions [30]. Here, we evaluate the population-level COVID-19 health burden and healthcare resource utilization associated with alternative school-based mitigation measures in a setting with strict physical distancing measures in the community and low population immunity. To this aim, we use an individual-based model (IBM) informed, calibrated, and validated with a diverse set of epidemiological data and estimates specific to the study area.

## Methods

### Data

This study was based on data from the Italian national COVID-19 Integrated Surveillance System, described elsewhere [31], and on validated analyses of contact tracing data [19, 28] in the province of Reggio Emilia (527 000 inhabitants, NUTS3 level) over the period 1 March–30 April 2021, when SARS-CoV-2 circulation was dominated by the Alpha variant.

We considered a line-list of 10 969 cases diagnosed within the province of Reggio Emilia with date of diagnosis or date of symptom onset within the study period, of which 6300 were symptomatic, 865 were hospitalized, 80 were admitted to an intensive care unit (ICU), and 130 died. The line-list contained individual-level information on the dates of diagnosis, symptom onset, and admission to hospital or ICU.

### Model

**Figure 1:**
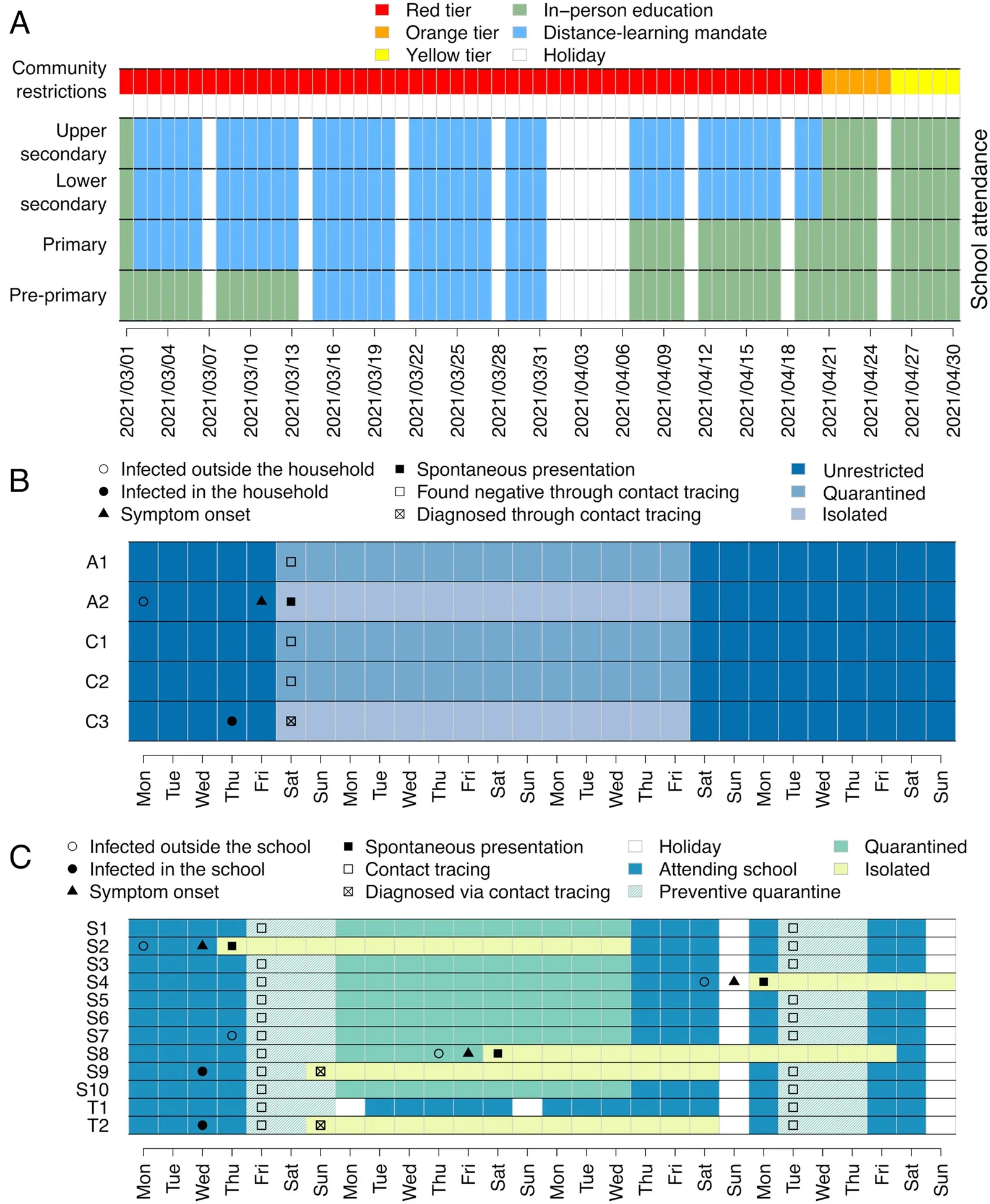
Summary of main physical distancing measures in place during 1 March–30 April 2021, in the province of Reggio Emilia, Italy. (A) Bundles of physical distancing measures applied to the general community were aggregated in progressively restrictive tiers (yellow tier: least restricted; red tier: most restricted [34]). Distance learning was mandated for all schools and classes of selected educational levels at various times during the study period. Sundays and Easter holidays, as per the official calendar, are represented in white. (B) An example of reactive interventions adopted in households, shown over a generic 4-week period. Individuals are shown as adults (A1, A2) or children (C1, C2, C3). The household index case (A2) is infected on Monday and transmits the infection to C3 on Thursday, while all other household members remain uninfected. A2 develops symptoms on Friday and spontaneously presents for diagnosis on Saturday, triggering their isolation, the testing of all household members on the same day via contact tracing, and a household quarantine for 14 days. Contact tracing triggers the diagnosis and isolation of C3, who remains asymptomatic. If C3 attended school before the household quarantine, a school contact tracing started as described in panel C. (C) An example of school-based reactive measures, shown over a generic 4-week period. Individuals are shown as students (S1, S2, ..., S10) or teachers (T1, T2). The class index case is a student (S2), who gets infected on Monday; transmits the infection to S9 and T2 and develops symptoms on Wednesday; spontaneously presents for diagnosis on Thursday. The diagnosis of S2 triggers contact tracing in their class, resulting in a 3-day preventive quarantine, so that all classmates and teachers can get tested and receive a result; because at least another case is found positive (in this case, S9 and T2), the quarantine of all classmates is prolonged for 10 additional days. Additionally identified cases are isolated at home. Other classes of T2 are set on distance learning due to teacher isolation as well (see an example in the Supplementary Text). T1, on the other hand, tests negative and can continue to teach in other classes. S7 was infected outside the class on Thursday, but tests negative at class contact tracing and remains asymptomatic: they will continue to infect in their household and in the community. S8 gets infected (outside the class) while their class is on quarantine and gets diagnosed 2 days later: they will not go back to class at the end of the class quarantine but only at the end of their isolation period. S4 gets infected (outside the class) on the first Saturday after the end of their class quarantine; they are diagnosed and isolated on Monday, triggering a new preventive class quarantine of 3 days and a new class contact tracing; because no other case is found in the class, in-person education can reprise after the 3 days.

We adapted an existing individual-based socio-demographic model [32] to SARS-CoV-2 transmission dynamics and control interventions in the study setting and period. We incorporated the school calendar, accounting for Sundays and Easter holidays [19, 28], and distance learning mandates in the study period (Fig. 1A). Individuals could transmit SARS-CoV-2 infection within households, schools, and the general community, according to a time-varying infectiousness [33], develop symptoms according to their age [13], and possibly seek healthcare assistance. Upon diagnosis, the patient was isolated and a 14-day quarantine was imposed on their household and school contacts (where applicable; see Fig. 1B and C and Supplementary Text). Other physical distancing bundle measures implemented at the community level during the study period were implicitly represented within the estimated community transmission rate.

### Calibration

To calibrate the free model parameters (the three transmission rates in households, schools and the general community, plus the number of initially infectious individuals), we applied Approximate Bayesian Computation based on Sequential Monte Carlo (ABC-SMC) [35], using as a score function the mean absolute percentage error across three components: (a) the distribution of the number of diagnosed cases in a household, derived from household contact tracing data [19, 33]; (b) the mean number of secondary cases caused in schools by infected individuals, previously estimated from an analysis of school contact tracing data [28]; and (c) the number of diagnosed symptomatic cases by week of symptom onset, obtained from surveillance data.

### Scenarios

The calibrated model, encompassing all the interventions implemented in the region over the study period, implements a baseline scenario referred to as scenario 1. We simulated counterfactual scenarios considering different school-based measures: scenario 0, where mandatory distance learning is imposed for the whole study period; scenario 2, where in-person attendance is allowed at all times, and school-based reactive measures are active; scenario 3 with no school-based measures in place; and scenario 4, which extended scenario 2 with an additional weekly universal screening of the student and teacher population, performed with salivary tests (80% sensitivity, 99% specificity) to be confirmed, if positive, by polymerase chain reaction (PCR) on the same day of screening. We ran a sensitivity analysis on the effectiveness of universal screening in schools, using a screening frequency of 14 days and different combinations of the test sensitivity and specificity. Results for all scenarios are presented by pooling together 100 stochastic replicates for each of 100 samples without replacement from the joint posterior distribution of parameters (total 10 000 simulations for each scenario).

### Model outcomes

We computed the initial reproduction number between 15 March and 22 March 2021, chosen to avoid both initialization effects and potential effects of saturation of susceptible individuals. The number of hospitalizations, ICU admissions and deaths associated with infections that occurred in the study period were computed by multiplying the cumulative attack rate by the infection hospitalization ratio (IHR), infection ICU ratio (IIR), and infection fatality ratio (IFR) previously estimated for the Alpha wave in Italy [36]. The peak hospital and ICU occupancies were computed considering the time series of simulated infections and the delay distributions for the incubation period [33], the time from symptoms to hospitalization or ICU admission (estimated from surveillance data), and the lengths of stay in the hospital and ICU [34, 37, 38]. Full details for model specifications, calibration, and processing of the outcomes are reported in the Supplementary Text.

## Results

**Figure 2:**
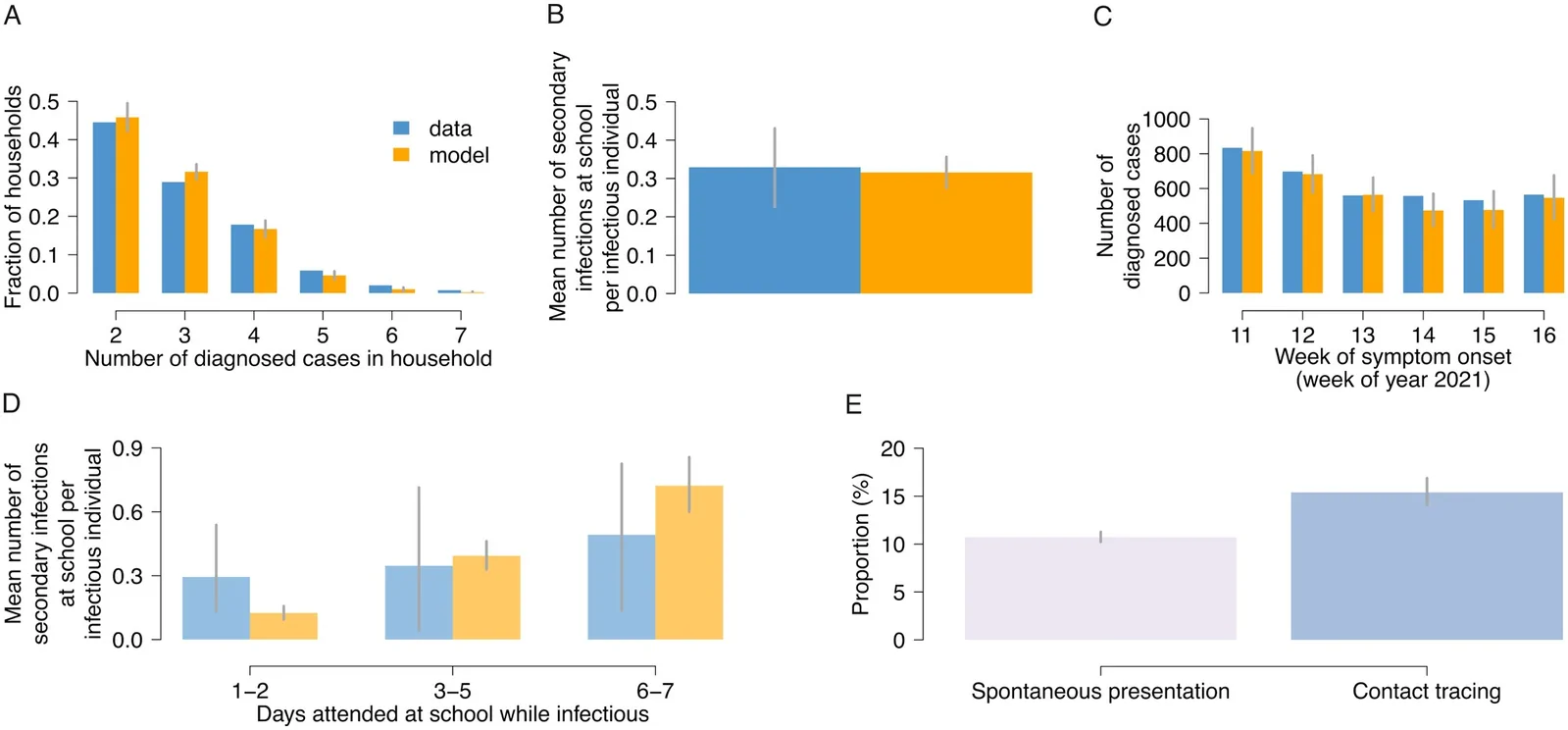
Model calibration and validation. (A) Distribution of the number of diagnosed secondary cases in households [19, 33]. (B) Mean number of secondary infections generated in schools by a single infector [28]. (C) Number of diagnosed cases by week of symptom onset (from the national COVID-19 surveillance system). (D) Mean number of secondary infections generated in the school setting by duration of school attendance before isolation of the infector [28]. (E) Proportion of ascertained infections by type of diagnosis (the remainder proportion was undiagnosed).

The model reproduced the distribution of the number of diagnosed cases in a household, the mean number of secondary cases generated in schools by infected individuals, and the number of diagnosed symptomatic cases by week of symptom onset [19, 28, 33] (Fig. 2A–C). The model also adequately reproduced secondary transmissions in schools stratified by the number of attendance days of the infector [28], information not provided during calibration (Fig. 2D). The model estimated that 26.1% (95% prediction interval [PrI]: 24.7%–27.7%) of all infections were detected by the surveillance system (over half of which through contact tracing, see Fig. 2E), consistently with a previous independent estimate of 23% for the infection ascertainment ratio in Italy during the same period [36].

**Figure 3:**
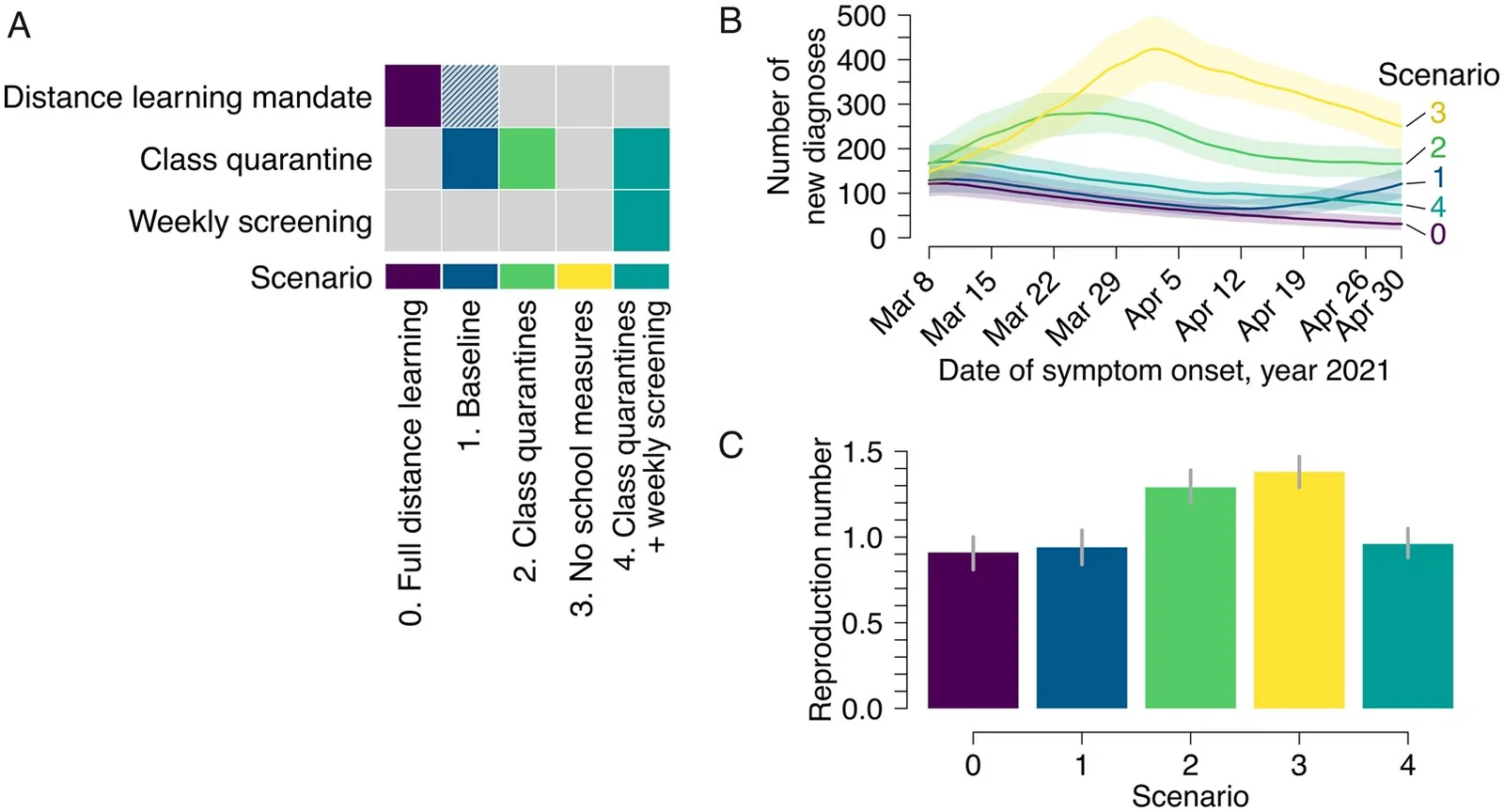
Effect of alternative school-based intervention scenarios on transmission. (A) Characteristics of the considered scenarios, where gray encodes inactive features and full color means active features; for scenario 1, distance learning mandate is represented by a shaded area to indicate that it was partially active over time, as reported in Fig. 1A. (B) Daily number of diagnosed cases by date of symptom onset. Colors correspond to scenarios reported in panel (A) of this figure, and scenario numbers are reported at the end of the curves for ease of reading. (C) Mean reproduction number estimated in the initial phase of the study period.

In scenario 1 (baseline), the number of new diagnosed cases by date of symptom onset declined slowly before rising again around mid-April 2021, as in-person education was progressively restored (Fig. 3B); in contrast, in scenario 0, cases kept declining until the end of the study period. In scenarios 2 and 3, where in-person attendance was maintained throughout the study period, cases increased rapidly before peaking and declining due to the saturation of the susceptible population. The only scenario with full in-person attendance that did not lead to a period of epidemic growth was the one with weekly universal screening (scenario 4).

Scenarios 0, 1, and 4 were associated with a mean initial reproduction number below the epidemic threshold (Fig. 3C): 0.91 (95% confidence interval [CI]: 0.81–1.00), 0.94 (95% CI: 0.84–1.03), and 0.95 (95% CI: 0.87–1.04), respectively. Scenarios 2 and 3, on the other hand, showed an initial reproduction number markedly above threshold, ranging from 1.29 (95% CI: 1.20–1.39) to 1.38 (95% CI: 1.29–1.47). The difference in reproduction numbers between scenarios 3 and 1 implies a contribution of approximately 0.47 (95% CrI: 0.37–0.58) from school transmission in the absence of interventions.

**Figure 4:**
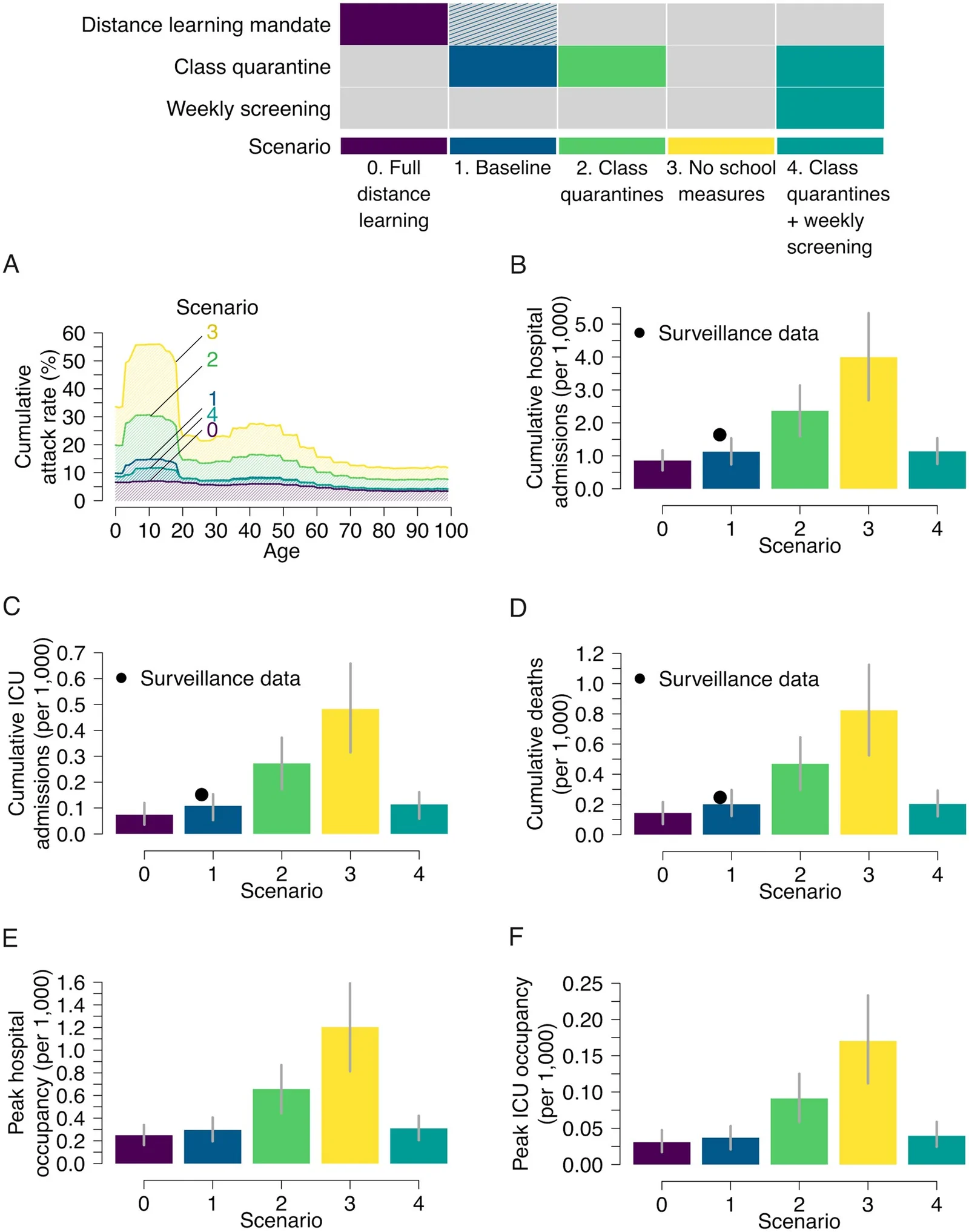
Effect of alternative school-based intervention scenarios on disease burden associated with infections occurring over the study period. (A) Cumulative attack rate by age. Scenario numbers are placed in correspondence of each curve for ease of reading. (B) Cumulative hospitalizations. (C) Cumulative intensive care unit (ICU) admissions. (D) Cumulative deaths. Points next to the scenario 1 bars in panels (B)–(D) represent the actual value observed in the Reggio Emilia province during the study period according to surveillance data and constitute a further validation of the model. (E) Maximum daily occupancy in hospital. (F) Maximum daily ICU occupancy.

In scenario 1 (baseline), cumulative infections were higher in school-aged individuals (5–18 years old) compared to scenario 0, due to in-person school attendance in the final part of the study period; however, age groups corresponding to typical parenting ages were also affected. In scenarios 2 and 3, the cumulative attack rate increased in all ages, reaching 28.5% and 51.9%, respectively, in student age groups (compared to approximately 13.8% in the baseline), and 7.6% and 11.8%, respectively, among individuals aged above 75 years (compared to 4.2%). These results are directly reflected in the burden of COVID-19-associated hospitalizations, ICU admissions, and deaths (Fig. 4B–D), as well as on peak hospital and ICU occupancy (Fig. 4E and F). Compared to scenario 1, approximately 4-fold values for all burden indicators were estimated if in-person school attendance was not mitigated by specific control measures (scenario 3), reducing to about 2.3-fold in the presence of reactive interventions (scenario 2). Weekly universal screening in schools (scenario 4) would result in a similar cumulative attack rate and COVID-19 burden as scenario 1, with the advantage of maintaining in-person attendance throughout the study period.

**Figure 5:**
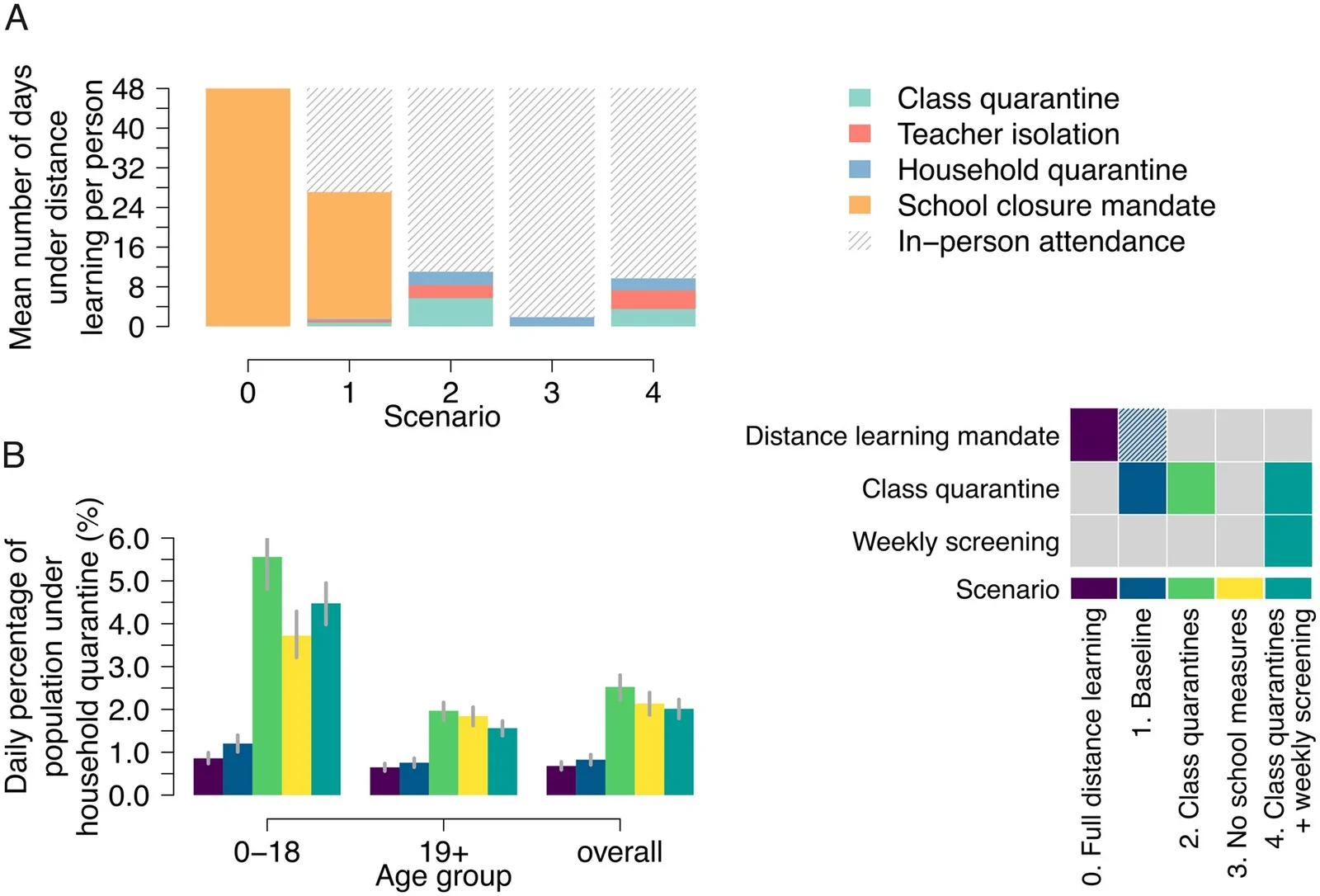
Effect of alternative school-based measures on in-person attendance and household quarantines. (A) Mean number of calendar school days per student, disaggregated by in-person attendance and by reason of missed attendance. (B) Mean proportion of population in household quarantine among under-age individuals, adults, and overall.

Of the 48 school days scheduled in the official school calendar during the study period, students attended an average of 21 days in person, primarily due to distance learning mandates (Figs 1A and 5A, scenario 1). Even without distance learning mandates, students would still have lost about 11.0 in-person school days (95% PrI: 10.0–12.1) in scenario 2 due to frequent household and class quarantines and teacher isolations associated with intense circulation of SARS-CoV-2 (Fig. 5A). Despite more intense case finding in scenario 4, which would be expected to result in more frequent household and class quarantines, the lower viral circulation would nonetheless result in fewer in-person days lost (9.7 days, 95% PrI: 8.7–10.8) than in scenario 2 (Fig. 5A). The mean fraction of the population in household quarantine (Fig. 5B) was low (around 0.8% per day) and similar across age categories in scenarios with distance learning mandates; in scenarios with full in-person attendance, this indicator was substantially higher among school-aged individuals (between 3.1 and 6.2% per day) and also higher among adults (between 1.3 and 2.1% per day).

**Figure 6:**
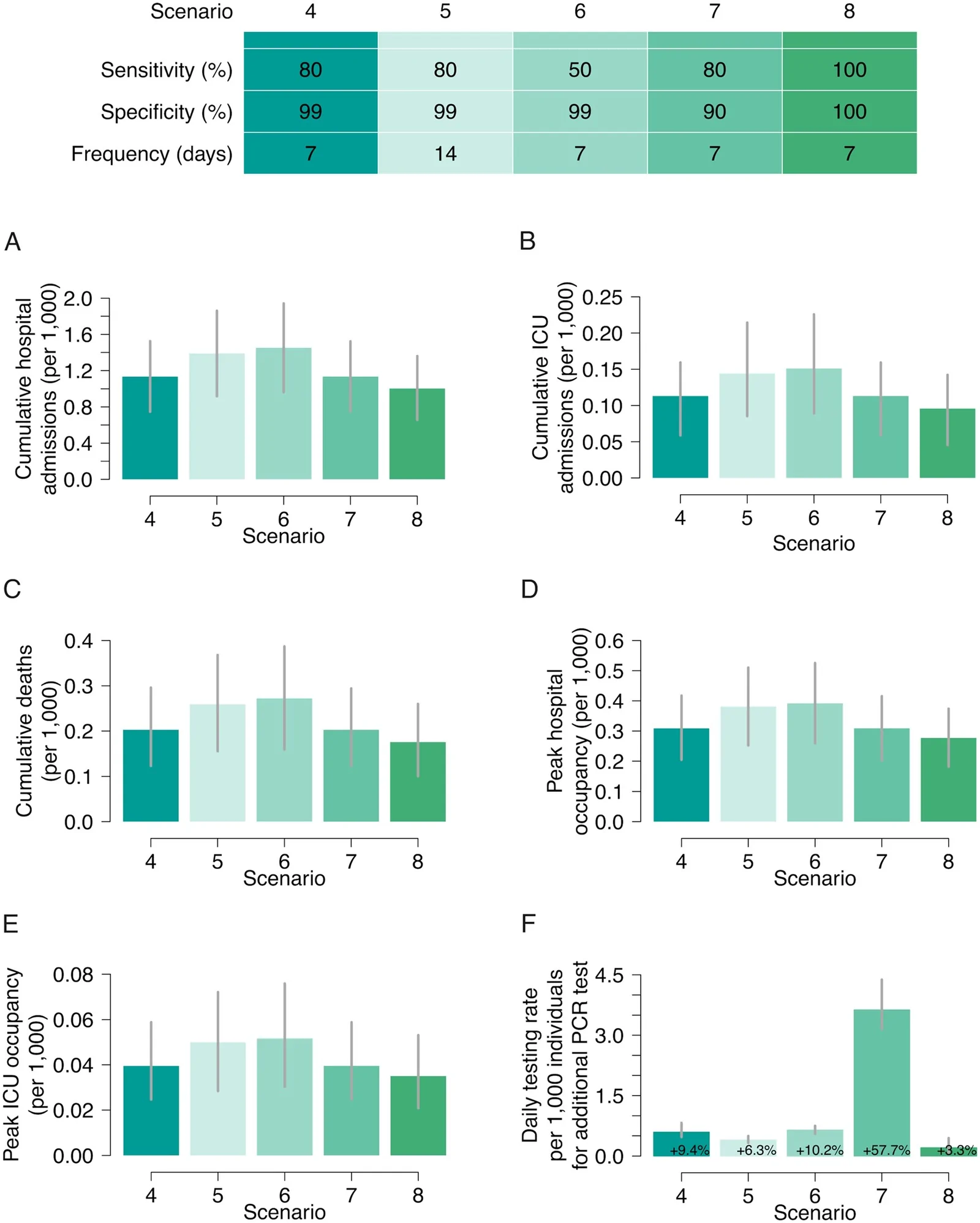
Effect of alternative screening frequency and performance parameters of the salivary screening test on disease burden and laboratory load. (A) Cumulative hospitalizations. (B) Cumulative intensive care unit (ICU) admissions. (C) Cumulative deaths. Points next to the scenario 1 bars in panels (B)–(D) represent the actual value observed in the Reggio Emilia province during the study period according to surveillance data and constitute a further validation of the model. (D) Maximum daily occupancy in hospital. (E) Maximum daily ICU occupancy. (F) Additional laboratory testing load due to confirmatory polymerase chain reaction (PCR) tests for positive screening tests; numbers at the base of the bars represent the relative increase with respect to the 6.3 tests per 1000 population per day actually performed in the study area and period [39].

The effectiveness of universal screening depended in part on screening frequency and test performance (Fig. 6). Reducing the frequency to fortnightly (scenario 5) would result in a COVID-19 burden comparable to that estimated using a salivary test with 50% sensitivity (scenario 6), with an estimated increase of about 20%–30% in health burden indicators relative to the baseline. Using a test with a lower specificity (90%, scenario 7) would keep the same effectiveness as scenario 4 but require 3.6 (95% CI: 3.1–4.4) confirmatory PCR tests per 1000 population per day (Fig. 6F), an almost 60% increase relative to the rate observed in the study period (6.3 per 1000 population per day [39]). By comparison, scenarios with 99% specificity show an increase in PCR testing burden of 10% or less. Perfect testing (scenario 8) would result in a slightly lower COVID-19 burden and in only a 3% increase in confirmatory tests.

## Discussion

We investigated the impact of school-based control measures on the transmissibility of SARS-CoV-2, the COVID-19 health burden, and the average in-person school days per student, using detailed epidemiological data and estimates from the Italian province of Reggio Emilia for the calibration and validation of a socio-demographic IBM. In the absence of school-based control measures, we estimated an increase of 0.47 units in the reproduction number associated with in-person attendance and a 4-fold COVID-19 morbidity and mortality, compared to what was observed in the study period. Despite the strict interventions existing at the time in the general population, at least 20% of infections were attributed to community transmission (Supplementary Text), allowing infections acquired by school-aged students to spill over to other age groups more subject to severe disease. The model was validated by the independent prediction of quantities not used during calibration, including the heterogeneity of transmission rates in schools, the infection ascertainment ratio, and the cumulative hospitalizations, ICU admissions, and deaths over the study period.

We also found that a weekly universal screening of the student and teacher population in addition to the implemented measures could eliminate the need for distance learning mandates while limiting the disease burden and hospital resource utilization. We note that a universal school screening based on nasal swabs (the testing technology available in early 2021) would have a poor acceptability for the student population and would have posed insurmountable logistical challenges for public health, due to the invasive specimen-collection procedure that needs to be performed by trained staff [40]. The subsequent introduction of saliva-based tests (since September 2021 in Italy [41]) removed both limitations. Weekly universal screening would not cause a higher loss of in-person days per student, although it would result in more days in household quarantine for adult individuals. While this might appear problematic for work absenteeism, this issue would be largely mitigated by the reduced need for parents to stay at home for childcare during distance learning mandates. Owing to the good specificity of SARS-CoV-2 salivary tests [42, 43], the additional logistical burden of confirming screening-positive results via PCR would entail an increase of less than 10% for the region’s laboratory effort during the study period [39]. However, both the epidemiological effectiveness and the additional laboratory effort were strongly dependent on the performance characteristics of the tests. During an emergency, estimates of these parameters for different products may require some time before a consensus is reached, potentially complicating decisions regarding the implementation of periodic school screening in a future pandemic.

Despite the limited geographical and temporal scope of the study, our results allow us to reason about the role of school transmission under other conditions. In our case, the low population immunity likely favored the spillover of infection from school-age to more vulnerable population segments. The estimated cumulative incidence of SARS-CoV-2 was below 10% in Italy at the end of February 2021 [36, 44], and only 7% of the population was fully vaccinated by the end of April 2021 [45]. Conversely, the strict community restrictions likely limited onward spread of infection to other age groups, thereby mitigating the contribution of school transmission to the population health burden. For over 90% of the study period, the considered population was under the “red” or “orange” tier, which corresponded to a 45%–55% reduction of visits to public venues, compared to pre-pandemic values [34]. Community restrictions in the red tier included a stay-home mandate (excluding work and health reasons), the closure of all shops (excluding essential retail/services), and the closure of food services (excluding takeaway and delivery), representing the most stringent measures adopted in Italy after the lockdown [34].

We acknowledge several limitations that may affect the reported results. The true potential contribution of schools to SARS-CoV-2 transmissibility may have been underestimated due to the presence of endogenous risk mitigation processes not accounted for in the model (e.g. face masks, hand hygiene, etc.). We did not consider dispersion in individual transmission potential. Since the impact of contact tracing is estimated to be higher under higher heterogeneity [46], the model would require higher transmission rates in schools and households to produce the observed number of infections, resulting in an amplified effect of unmitigated school transmission and relaxing the constraints on the sensitivity of the screening test performance. The model does not explicitly represent aerosol transmission, whose contribution to pathogen spread may be reduced by improved air ventilation [2], potentially providing additional means to support the maintenance of in-person education alongside periodic screening. We did not consider the potential effect of the decreased susceptibility in children, which was demonstrated for ancestral SARS-CoV-2 lineages [11], but not for subsequent variants. Our choice is supported by the comparison of model results on the estimated number of secondary cases by educational levels (Supplementary Text): when a higher susceptibility is assumed in young adults compared to children up to 15 years of age, a substantially higher number of secondary cases is expected within high schools compared to other school levels, which contrasts with the available evidence for our study population [28]. Note also that the assumption of a reduced relative susceptibility in children does not necessarily translate into a decreased impact of in-person school attendance on population transmission: in such a scenario, the calibrated transmission rates in schools would need to be higher to reproduce the same data. Finally, teachers were a priority group for vaccination due to their occupational exposure; their vaccine coverage may therefore have been substantial at least in a part of the study period; however, we did not consider vaccination among teachers due to a lack of specific longitudinal coverage data for this group.

Our results suggest that, in the presence of a respiratory infection with high transmissibility, high rates of underreporting and large population susceptibility, distance learning mandates may be a necessary evil to protect vulnerable population segments and substantially reduce the burden of disease. However, combining periodic screening in schools with reactive class quarantines may safeguard in-person education without overburdening healthcare and laboratory facilities, provided that screening tests with high specificity are available. These findings may support the assessment of school-related control measures during future emerging respiratory infections and particularly during pandemics. We stress that specific quantitative evaluations depend on the epidemiological characteristics of the pathogen (e.g. transmissibility of asymptomatic individuals, severity, and fatality), the proportion of infections identified by surveillance systems, and the age-heterogeneity of these parameters, combined with the population age structure. This study demonstrates that the analysis of high-quality contact tracing data in schools and households, combined with standard surveillance data, provides sufficient information to assess the population-level impact of transmission in educational settings and the effectiveness of associated control measures. In the case of a new pandemic threat posed by a respiratory pathogen, the prompt collection of such data is therefore of primary importance for an appropriate response that minimizes both the epidemiological and the societal harms caused by the suspension of in-person education.

## Ethics approval

The collection of data used for this manuscript (surveillance and contact-tracing data) is compulsory in Italy according to national laws on infectious diseases. The COVID-19 Italian National Working group on Bioethics has stated that consensus for the collection of this data in the context of the COVID-19 emergency was not mandatory (Rapporto ISS COVID-19 n. 34/2020), based on Guideline 12 of the WHO on ethical issues in public health surveillance. The legal ordinance n. 640 of 28 February 2020, explicitly declares Istituto Superiore di Sanità as entitled to collect data for COVID-19 surveillance and contact tracing and that such data can be used and shared, upon anonymization, to advance scientific knowledge on this new disease.

## Supplementary Material

dyag103_Supplementary_Data

## Data Availability

Data and software underlying this article are available on Zenodo (https://doi.org/10.5281/zenodo.20703516).
