# Supplementary material for: The impact of school-based mitigation measures on the transmission of respiratory pathogens: the case of SARS-CoV-2
Source: Int J Epidemiol. 2026 Jul 9;55(4):dyag103. doi: 10.1093/ije/dyag103 (PMC13348709; doi:10.1093/ije/dyag103)
Supplement: dyag103_Supplementary_Data [file dyag103_supplementary_data.pdf]

Supplementary Material

The impact of school-based mitigation measures on the transmission of respiratory pathogens: the case of SARS-CoV-2

Laura Fumanelli[1,2], Giorgio Guzzetta[1], Martina del Manso[3], Antonino Bella[3], Massimo Vicentini[4], Olivera Djuric[4,5], Eufemia Bisaccia[6], Pamela Mancuso[4], Silvia Cilloni[6], Elisabetta Larosa[6], Patrizio Pezzotti[3], Paolo Giorgi Rossi[4], Stefano Merler[1,*].

1. Center for Health Emergencies, Bruno Kessler Foundation, Trento, Italy
2. DONDENA Centre for Research on Social Dynamics and Public Policy, Bocconi University, Milan, Italy
3. Department of Infectious Diseases, Istituto Superiore di Sanità, Rome, Italy
4. Epidemiology Unit, Azienda Unità Sanitaria Locale – IRCCS di Reggio Emilia, Reggio Emilia, Italy
5. Centre for Biostatistics, Epidemiology, and Public Health (C-BEPH), Department of Clinical and Biological Sciences, University of Turin, Turin, Italy
6. Public Health Unit, Azienda Unità Sanitaria Locale – IRCCS di Reggio Emilia, Reggio Emilia, Italy

*corresponding author: merler@fbk.eu

Table of contents:Supplementary Material 1
1. Supplementary Methods 2
1.1 Study period and data 2
1.2 Socio-demographic Individual-Based Model 2
1.3 Transmission model 4
1.4 Case diagnosis 6
1.4.1 Spontaneous presentation 6
1.4.2 Contact tracing in households 7
1.4.3 Contact tracing in schools 7
1.5 Physical distancing measures 8
1.6 Initialization 9
1.6.1 Initial prevalence by age group and time since infection 9
1.6.2 Initial immune population 9
1.7 Calibration 10
1.7.1 Free model parameters 10

1.7.2 ABC-SMC 10
1.8 Model outcomes 11
1.9 Scenarios 11
2. Additional results 12
2.1 Supplementary results for baseline and counterfactual scenarios 12
2.2 Susceptibility by age 13
References 14

1. Supplementary Methods

1.1 Study period and data

We focused on the province of Reggio Emilia, Italy, where the availability of high-quality contact tracing data allowed the previous estimation of epidemiological quantities which could be used in this study for model calibration [1,2]. These quantities were estimated relative to the period March 1 – April 30, 2021, during which, according to genomic survey data, the Alpha variant was predominant in the considered population (87% in mid-March, 93.2% in mid-April) [3]. Thus, the study period for our transmission model was inherited by the previous studies. In particular, we used estimates of the incubation period and generation time distributions for the Alpha variant [1], and the distributions of household clusters by size of the cluster, starting from data on 9724 SARS-CoV-2 cases clustered in 3545 households where at least one secondary case was recorded [4]. In addition, we used estimates of the number of secondary cases transmitted in educational settings [2], derived from 284 SARS-CoV-2 confirmed positive individuals among students and school personnel from 87 school outbreak investigations.

1.2 Socio-demographic Individual-Based Model

Building on previously developed Individual-Based Models (IBM) [5-8], we generated a synthetic population for the province of Reggio Emilia, Italy, reproducing detailed socio-demographic data on age structure, household size and composition, employment rates by age, and the educational system [9-14] (see Figures S1-S4 for a validation of the socio-demographic model). The detailed procedure for the generation of the synthetic population and further validation of the socio-demographic structures can be found in previously published work [7,8].

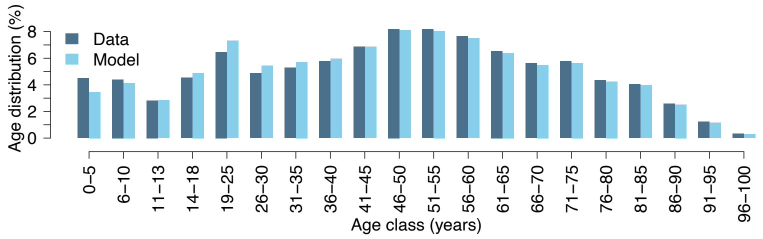

Figure S1. Comparison between the empirical and modeled population age structure. Age classes in children are grouped by school ages (pre-primary: less than 5 years old; primary: 6-10; lower secondary: 11-13; upper secondary: 14-18); except for the age class 19-25, all other age classes are grouped by 5-years brackets.

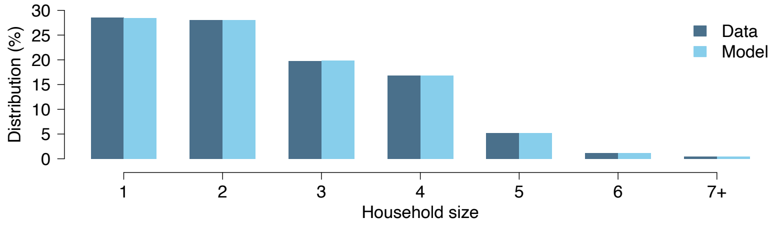

Figure S2. Comparison between the empirical and modeled household size distribution.

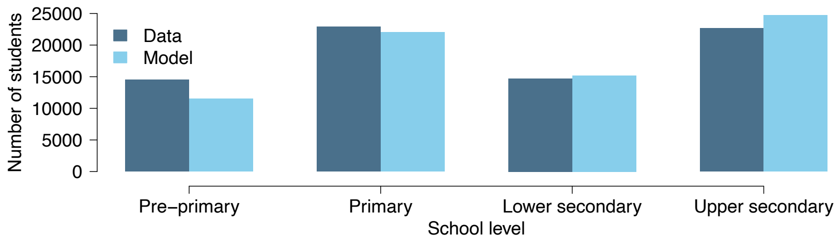

Figure S3. Comparison between the empirical and modeled student population by educational level.

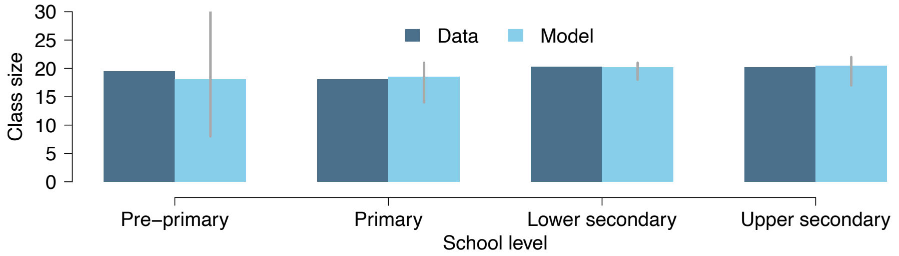

Figure S4. Comparison between the empirical and modeled average class size by educational level.

We considered the population of the province of Reggio Emilia (about 527,000 inhabitants), distributed over 150 georeferenced cells, based on data from the Gridded Population of the World version 4 [9] and administrative layers for the study area [15]. Every individual of the population was assigned an age and associated to a specific household. We used a heuristic model matching marginal distributions of household size, household age by size, age of household members by size (and thus the age structure of the total population), and maintaining realistic generational age gaps within household members. We did not consider non-private households or families with aggregated members. All households with more than four members were assumed to

be composed by a couple with children, since type “couple with children” represents more than 95% of the total number of households [10]. Overall, simulated households complied very well with real data, in terms of average size and distribution of age groups given the size and type of composition: this supports the validity of our choices on household types.

Every individual was then assigned to either a school (as a student or teacher depending on age) according to data on occupation by age [10-12].
Four educational levels have been considered, in agreement with the educational structure of Italy [13]: a) pre-primary education (daycare centers and preschools) up to 5 years old; b) primary school for children from 6 to 10 years old; c) lower secondary school from 11 to 13 years old; and d) upper secondary school from 14 to 18 years old. All children in compulsory schooling age (6-16 years-old) were assigned to an educational level depending on their age; for all other ages, assignment to schools was based on age-specific census data [10-12]. Students older than 18 were considered to attend higher education settings (post-secondary training, universities and doctoral programs) but these were not explicitly included in transmission. A proportion of adults in the model were employed as teachers according to age-specific data [10].
For each school level the corresponding information on school size distribution has been taken into account in order to obtain a realistic size [14,16]; in addition, all schools from pre-primary to upper secondary education were further partitioned into classes according to the average class size by educational level in Italy: 18 pupils per class in primary education and 20 pupils per class in pre-primary and secondary education [12,17]. Pupils belonging to the same class have the same age in primary and secondary school levels; in pre-primary schools we implemented classes of mixed age for pupils aged 0-2, and same-age classes for pupils aged 3-5, reflecting the typical organization of pre-primary education in Italy [16]. Teachers were assigned to schools in such a way to match the pupil-to-teacher ratio in every educational level [17]; each teacher was assigned to three different classes of the same school, resulting in an average number of teachers per class ranging from 5 in pre-primary schools to 6 in upper secondary schools.

We incorporated in the model the calendar for the 2020/21 school year that was established by the regional scholastic department of Emilia-Romagna [18], to which educational institutes in the Reggio Emilia province referred.

1.3 Transmission model

Transmission of SARS-CoV-2 between individuals could occur in three different settings, namely household, schools, and the general community [6-8], according to setting-specific transmission rates.
At any time t, an infectious individual i could infect other members of the household or school with a binomial probability $p_i(t) = 1 - \exp\left(-\lambda_{i,L}(t)\Delta t\right)$, where $\lambda_{i,L}(t)$ is the force of infection exerted at time t by individual i in setting $L \in \{H, S\}$. We assumed that $\lambda_{i,L}(t)$ varies over time according to the formula:

$$\lambda_{i,L}(t) = \beta_L\, k(t - \tau_i)/N_{i,L},$$

where

- β_L is the setting-specific transmission rate;
- $k(\sigma)$ is the relative infectivity of individual i, σ days since infection;
- τ_i is the time at which individual i was infected;
- $N_{i,L}$ is the number of individuals in the same setting L to which individual i belongs. For household transmission ($L = H$), $N_{i,H}$ is the household size; for school transmission ($L = S$), $N_{i,S}$ is the number of students and teachers belonging to that specific school who were not isolated, quarantined or under distance learning.

In the community setting, the force of infection was spatially heterogeneous. The number of new infections was determined by a Poisson distribution of mean

$$\mu = \left[1 - \exp\left(-\lambda_{i,R}(t)\right)\right] N$$

where N is the total number of individuals in the population, and transmission in the community setting is modeled as

$$\lambda_{i,R}(t) = \frac{\beta_R k(t - \tau_i) f(d_{ij})}{\sum_{j=1}^{N} f(d_{ij})}$$

where

- β_R is the transmission rate in the community;
- $k(\sigma)$ is the infectivity of individual i, σ days since infection;
- τ_i is the time at which individual i was infected;
- d_{ij} is the geographical distance between individuals i and j;
- f is a kernel function defined as

$$f(d) = (d + r_g^0)^{-\beta_m} e^{-d/\kappa}$$

with $r_g^0 = 5.8$ km, $\beta_m = 1.65$ and $\kappa = 350$ km [19, 20].

The relative time-varying infectiousness $k(t - \tau_i)$ was assumed to be proportional to the distribution of the intrinsic generation time, estimated for the SARS-CoV-2 Alpha variant, i.e. a discretized gamma distribution with shape 2.53 (95% CrI:2.27-3.21) and scale 2.83 (95% CrI: 2.28-3.39) [1], an offset of one day (implying that the individual can not transmit on the same day of their infectious episode), and censored at day 21 (i.e., no transmissibility after 21 days since infection) (Figure S5).

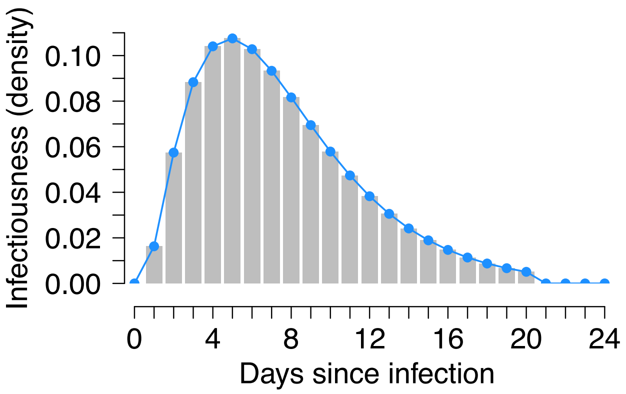

Figure S5. Assumed temporal evolution of an individual's relative infectiousness.

1.4 Case diagnosis

Case diagnosis could occur either via spontaneous presentation of symptomatic individuals, or through contact tracing in households and schools. All diagnosed individuals were required to undergo a 14-day isolation period, which prevented them from contributing to community and (if relevant) school transmission during that time.

1.4.1 Spontaneous presentation

Infected individuals could develop symptoms with an age-dependent probability [21] (Table S1), after an incubation period sampled from a gamma distribution with shape 3.08 (95% CI: 2.56-3.86) and scale 1.58 (95% CI: 1.24-1.93) [1].

Age group	Symptoms probability
0-19	18.1%
20-39	22.4%
40-59	30.5%
60-79	35.5%
80+	64.5%

Table S1. Probability of symptoms by age [21].

We assumed that a fraction of symptomatic individuals would spontaneously present for diagnosis, with a delay from symptom onset sampled from a diagnostic delay distribution estimated from data.

- The probability of spontaneous presentation by symptomatic individuals was estimated as follows: from the Italian line list of cases we selected 11,232 SARS-CoV-2 cases having a date of diagnosis comprised within the study period (March 1, 2021 – April 30, 2021) and whose residence was in the province of Reggio Emilia. Of these, 6638 were symptomatic.
- The proportion of symptomatic individuals who will be diagnosed α_s can be obtained from:

$$\alpha_s = \frac{\alpha}{\pi} P_s$$

Where α is the total infection ascertainment ratio, π is the average proportion of symptomatic infections and P_s is the fraction of symptomatic individuals in notified cases. Considering a previous estimate of $\alpha = 22.9\%$ for the study period at a national level [22], $\pi = 31\%$ [21], and $P_s = \frac{6638}{11232} = 59.1\%$, for the province of Reggio Emilia we obtained $\alpha_s = 43.4\%$.
Similarly, we can estimate the proportion of symptomatic individuals who will be diagnosed α_a as:

$$\alpha_a = \frac{\alpha}{(1-\pi)} (1 - P_s),$$

resulting in $\alpha_a = 13.6\%$. We assumed that the ascertainment of asymptomatic infection could only occur through contact tracing procedures, therefore we did not use this value for the model. However, we notice that the estimated proportion of infections ascertained through contact tracing in the main analysis was very close to this value, validating the approach.
- The diagnostic delay between symptom onset and diagnosis was computed by fitting a log-normal distribution to the cumulative proportion of non-negative

diagnostic delays (>90% of the 6638 symptomatic cases) obtained from the province-level dataset. We found a mean of 0.9711 and standard deviation 0.6257 for the resulting diagnostic delay distribution. The agreement with data is shown in Figure S6.

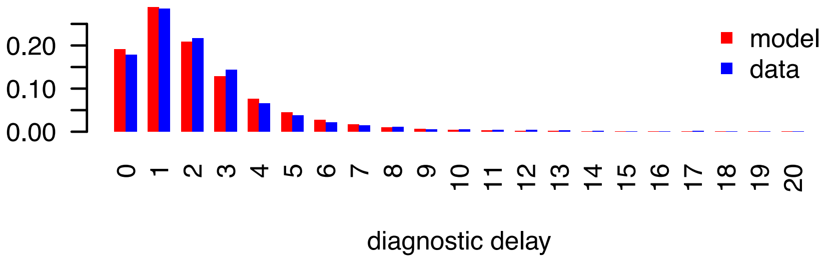

Figure S6. Comparison between the empirical and modeled distribution of the diagnostic delay (in days).

1.4.2 Contact tracing in households

Household members of diagnosed cases were subject to contact tracing, with immediate PCR testing, and quarantined for 14 days; at the end of this period, the entire household was tested again and an additional 7-day extension was imposed if any member tested positive on that occasion. We assumed that PCR testing had perfect sensitivity between the second and the fifteenth day from infection and that it would miss the infection otherwise.

At the beginning of a household quarantine, contact tracing was carried out for student and teacher household contacts to identify asymptomatic cases that were the actual source of the newly detected cases [23]. Specifically, household contacts of an index case, who were students or teachers and were found positive through household contact tracing, triggered school contact tracing as detailed below.

1.4.3 Contact tracing in schools

A reactive quarantine protocol was active during periods of in-person education.

- In primary and secondary (both lower and upper) schools, a diagnosis in a student mandated a preliminary three-day quarantine and contact tracing with immediate PCR testing for all school close contacts encountered within 48 hours before the diagnosis (i.e., classmates and teachers associated to the class) [23,24]. Finding an additional case among classmates resulted in a 10-day extension of class quarantine with a test at quarantine exit [4]. Teachers who were contacts of a case were not required to quarantine when showing a negative PCR test result (Figure 1 in the main text).
- In primary and secondary schools, as soon as a teacher tested positive for SARS-CoV-2, distance learning was imposed on all their students for 14 days without testing (Figure S7A).
- In pre-primary schools (daycare centers and preschools), a 14-day class quarantine was triggered automatically upon positivity of either a pupil or a teacher [4]. Immediate PCR testing was applied to all classmates and teachers associated to the class (Figure S7B).

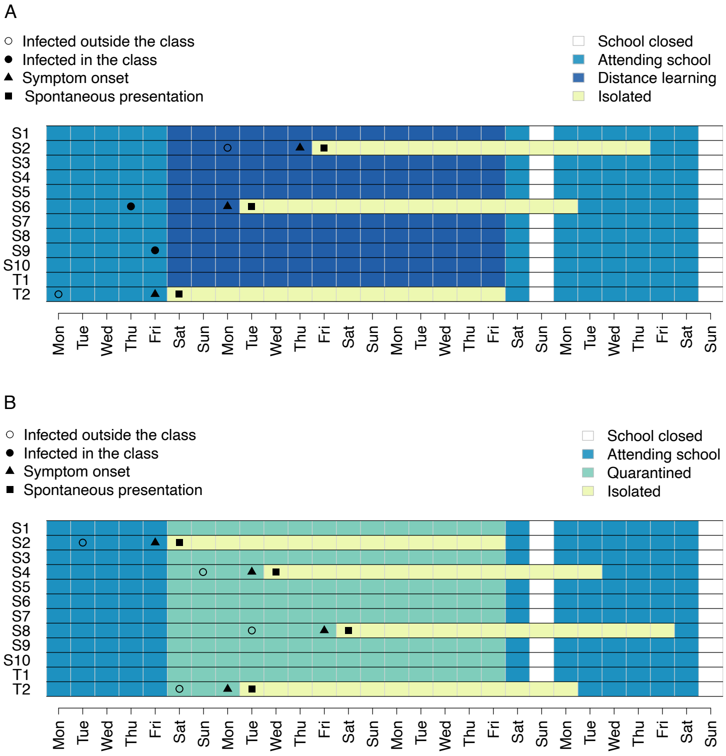

Figure S7. Example of school based reactive measures, shown over a generic 4-week period. Individuals are shown as students (S) or teachers (T). **A.** Any education level, except the pre-primary, when the class index case is a teacher. T2 gets infected on Monday; transmits the infection to S6 on Thursday and S9 on Friday; develops symptoms on Friday and spontaneously presents for diagnosis on Saturday. This triggers a 14-day isolation period for T2 and a 14-day distance learning period for all students of the class. S2 acquires infection while on distance learning. S2 and S6 show symptoms while on distance learning and are thus isolated for 14 days since spontaneous presentation. S9 never shows symptoms. **B.** Class at pre-primary level with a student index case (S2). S2 gets infected on Tuesday; develops symptoms on Friday and transmits the infection to S4 and T2 on the same day; spontaneously presents for diagnosis on Saturday. This triggers a 14-day quarantine for all classmates and teachers. S4 and T2 show symptoms during quarantine and are thus isolated for 14 days since spontaneous presentation. S8 gets infected and shows symptoms during quarantine and is thus isolated for 14 days starting from spontaneous presentation. S7 gets infected outside the class on Friday before the start of quarantine but never gets isolated as they never develop symptoms.

1.5 Physical distancing measures

Starting November 2020 the Italian government introduced a system of progressive restriction tiers according to real-time epidemiological risk assessments at the regional level [25]. These tiers were labeled according to a color scheme: yellow, orange, and red, corresponding to increasing levels of restrictions on school attendance, travels, shops and leisure activities. The measures were further refined in March 2021 [26] and made more restrictive in April 2021 [27]. Due to its epidemiological and healthcare system situation, the Emilia-Romagna region was subject to the red tier from the start of the study period until April 20, 2021; to the orange tier from April 21 to April 25, 2021; and to the yellow tier from April 26, 2021 to the end of the study period.

Within these provisions, distance learning was mandatory for all primary and secondary schools from March 2, 2021 to April 6, 2021 (April 11 for the upper secondary level), while daycare centers and preschools remained open until March 14, 2021 and were subject to distance learning from March 15 to April 6, 2021. School-based measures were explicitly implemented according to the calendar of distance learning mandates. The remaining restrictions to the general community were implicitly accounted within the estimate for the transmission rates in the community. In practice, we simply assume that the estimated transmission rate in the community would have been higher in absence of NPIs.

We assumed that household and community transmission rates remained unchanged during class quarantines and distance learning periods, and that individuals under isolation or quarantine (household or classroom) could transmit only to household members. Individuals in distance learning (either because of mandates or because of a diagnosed teacher) can transmit to household members and in the general community.

1.6 Initialization

1.6.1 Initial prevalence by age group and time since infection

We selected from the Italian line list of cases all symptomatic SARS-CoV-2 cases diagnosed in the province of Reggio Emilia having date of symptom onset comprised between 21 days earlier and 21 days after the beginning of the study period in March 1, 2021 (i.e., from February 8 to March 22, 2021). For each case, we assigned a date of infection by subtracting from the date of symptom onset an incubation period sampled from the distribution reported above, then we kept only those cases with imputed infection date between 1 and 20 days before March 1 (i.e., between February 8 and February 28). Symptomatic cases were grouped by date of infection and age-group (0-19, 20-39, 40-59, 60-79, 80+). The distribution of infections by date of infection and age group was obtained by multiplying the number of symptomatic cases by an age group-specific factor

$$M(a) = \frac{1}{p_s(a)\, \alpha_s\, \chi}$$

where $p_s(a)$ is the age-specific probability of developing symptoms, α_s is the previously estimated probability of reporting for symptomatic cases in Reggio Emilia and χ is a normalization factor so that the distribution by date of infection and age group would sum up to 1. The distribution was then rescaled by a free parameter representing the total number of infectious individuals at the beginning of the simulation (see section 1.7 below). For all initial individuals, the probability of symptom development, diagnosis and dates of diagnoses were sampled as specified in section 1.4, and household and class members were initialized as isolated or quarantined accordingly.

1.6.2 Initial immune population

We considered the cumulative incidence in the period before the Alpha variant became dominant (between February 21, 2020 to February 17, 2021) as estimated in [22], we

assumed that 14.2% of individuals had already been infected with SARS-CoV-2 and were thus randomly assigned as immune in the model.

1.7 Calibration

1.7.1 Free model parameters

The free model parameters were the three transmission rates in household (β_H), schools (β_S) and the general community (β_R), and the total number of initially infectious individuals (I_0), explored over broad value ranges: $\beta_H \in [0,9]$, $\beta_S \in [0,6.4]$, $\beta_R \in [0,6.4]$, $I_0 \in [2000,27000]$.

1.7.2 ABC-SMC

To calibrate the free model parameters, we applied Approximate Bayesian Computing based on Sequential Monte Carlo (ABC-SMC) [28]. We used as a score function the Mean Absolute Percentage Error:

$$MAPE(P) = \frac{\sum_{i=1}^{n} |A_i - P_i|}{\sum_{i=1}^{n} |A_i|}$$

where P_i is the predicted value, A_i the actual value, and n is the number of fitted components [29].

We computed MAPE for three components: (a) the distribution of the number of diagnosed cases in a household, deriving from household contact tracing data [1,4]; (b) the mean number of secondary cases caused in schools by infected individuals, previously estimated from an analysis of school contact tracing data [2]; and (c) the number of diagnosed symptomatic cases by week of symptom onset, obtained from surveillance data. We ran 100 stochastic simulations for each parameter set, and we considered as P_i the value given by the mean over all simulations. The final score for each parameter was given by the sum of the three MAPE values.

We performed two SMC steps, with 20,000 parameter sets sampled uniformly and independently from a broad range of values in the first step. In the second step, we applied to the top 100 parameter sets from the first step, 200 samples of a four-dimensional Gaussian perturbation kernel, obtaining 20,000 new parameter sets. The covariance matrix of the perturbation kernel was assumed to be diagonal (corresponding to independent perturbations on each parameter). The standard deviations for each free parameter (i.e., the elements of the diagonal of the covariance matrix) were set according to the same empirical rule:

- selected parameter values from the first ABC-SMC round are ranked in increasing order;
- distances between successive ranked values are computed;
- the standard deviation of the parameter-specific perturbation is set equal to the mean of the ranked distances.

We set an acceptance threshold for the MAPE such that the top 1,000 parameter sets of the second step would be selected, representing an approximation of the joint posterior distribution of parameters.

1.7.3 Posterior distributions

Marginal posterior distributions were well within the broad range of explored parameter values (set as ranges for the x axis in Figure S8). We then sampled without replacement 100 parameter sets from the joint posterior distributions and simulated scenarios by running 100 stochastic replicates for each parameter set (total 10,000 simulations for each scenario).

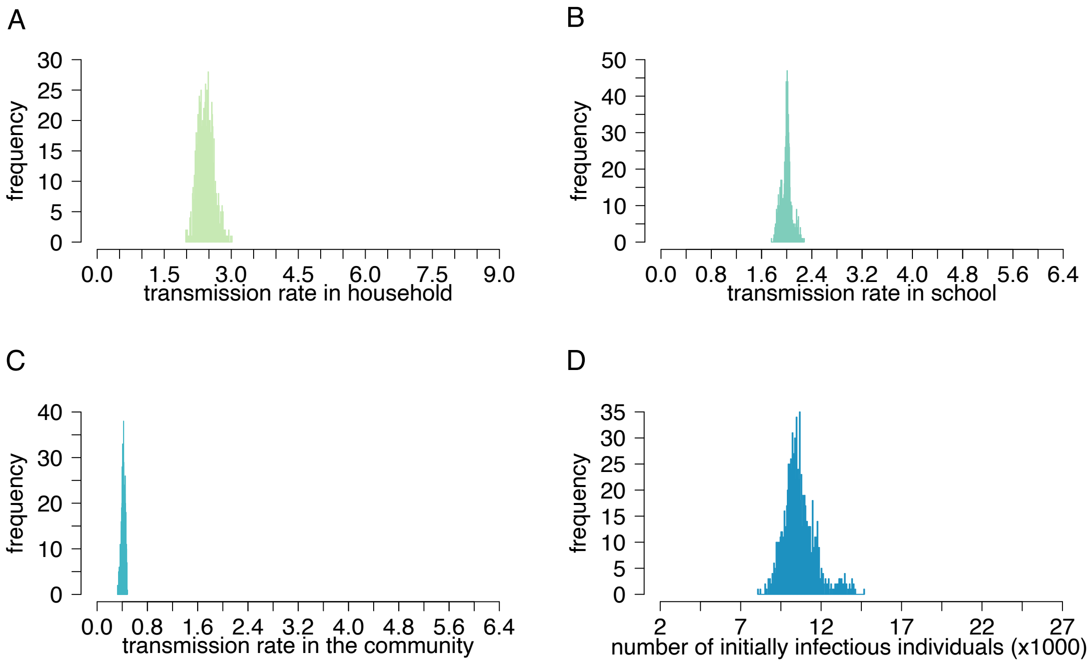

Figure S8. Approximations of the marginal posterior distributions for free model parameters. Ranges for the x axis correspond to the range of parameter values explored.

1.8 Model outcomes

The time series of hospital and ICU bed occupancy was computed by multiplying the time series of simulated infections in each scenario by previous estimates of the infection hospitalization ratio (IHR) and the infection ICU ratio (IIR) , respectively, computed for the Alpha wave in Italy [22], and then applying to such time series and the delay distributions for the incubation period [1], the time from symptoms to hospitalization or ICU admission (estimated from surveillance data), and the lengths of stay in the hospital and ICU [30-32]. The maximum value of such time series was reported as the peak hospital and ICU occupancy.

1.9 Scenarios

We implemented a baseline scenario, named “scenario 1”, accounting for all interventions and school-based restrictions that were adopted in the province of Reggio Emilia during the study period: school closure with mandatory distance learning as detailed in section 1.5, case isolation, household quarantine, household contact tracing, class quarantine, reactive distance learning due to teacher isolation, school contact tracing.

In addition, we considered four counterfactual scenarios:

- *Scenario 0*: all schools were closed throughout the entire study period;
- *Scenario 2*: schools followed the regular school calendar, and the same interventions as in the baseline were implemented;
- *Scenario 3*: schools followed the regular school calendar and no school measures in place;
- Scenario 4: as Scenario 2 but with periodic screening involving the entire school population. Students and teachers were tested every week with an antigenic test having 80% sensitivity and 99% specificity [33-35]; individuals who were found positive were then tested again with a PCR test on the same day. Classes having two or more students who were positive to PCR upon screening were quarantined for 14 days; daycare centers and preschools required only one student (or teacher) positive to PCR to quarantine the class.

We also performed a sensitivity analysis on the frequency and performance parameters of the periodic universal school screening, with the following scenarios:

- Scenario 5: as Scenario 4 but with 80% sensitivity, 99% specificity and 14-days frequency;
- Scenario 6: as Scenario 4 but with 50% sensitivity, 99% specificity and 7-days frequency;
- Scenario 7: as Scenario 4 but with 80% sensitivity, 90% specificity and 7-days frequency;
- Scenario 8: as Scenario 4 but with 100% sensitivity, 100% specificity and 7-days frequency.

1.10 Realizations and resources

The model was implemented in C language. Simulations were run on an Intel® Xeon® W-2155 CPU @ 3.30GHz with an x86_64 architecture. The simulation time scaled with the number of infections; for reference, 100 stochastic iterations of 100 parameter sets for the baseline scenario (one of the least computationally intensive scenarios) were run in parallel on 20 cores in 14 minutes.

2. Additional results

2.1 Supplementary results for baseline and counterfactual scenarios

We evaluated the proportion of cases by setting (household, school and community) and the overall mean number of secondary infections generated at school for the baseline and the counterfactual scenarios (Figure S9). Note that, even under the least stringent scenario 3, at least 20% of all infection episodes were attributed to community transmission.

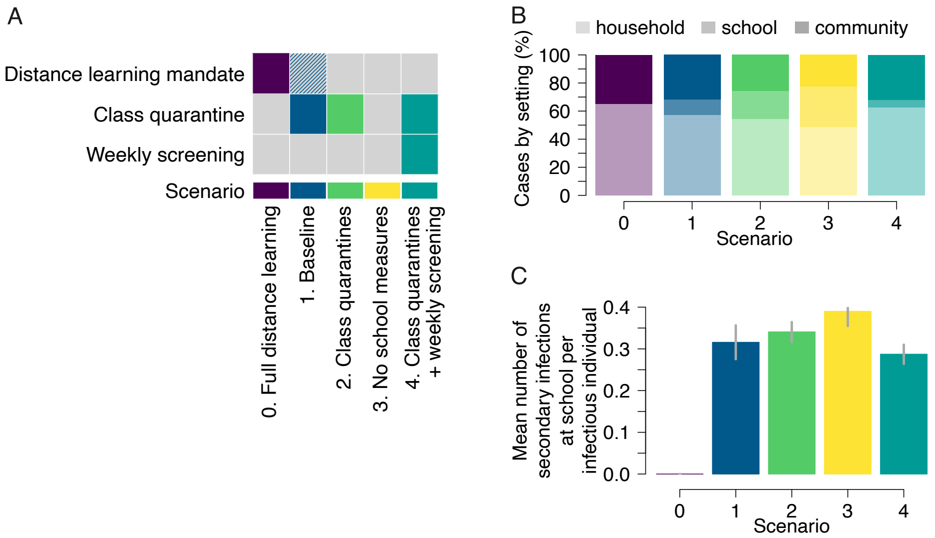

Figure S9. Additional results for the main scenarios. A. Characteristics of the considered scenarios, where gray encodes inactive features and full color means active features; for scenario 1, distance learning mandate is represented by a shaded area to indicate that it was partially active over time, as reported in Figure 1A of the main text. **B.** Proportion of cases by setting of transmission, which are shown in different color intensity. Colors correspond to scenarios reported in panel A of this figure. **C.** Mean overall number of secondary infections generated in the school setting before isolation of the infector.

2.2 Susceptibility by age

In Figure S10, we compare model-estimated secondary infections by educational levels with available estimates. When considering the model used in the main text, which assumed an age-homogeneous susceptibility, the comparison is quite accurate, providing further validation to the study (Figure S10A). However, when considering a reduced susceptibility in children aged 15 years or less, and an increased susceptibility in adults aged 65 years or more (panel B), as estimated for ancestral lineages [36], model validation is much less accurate (Figure S10B).

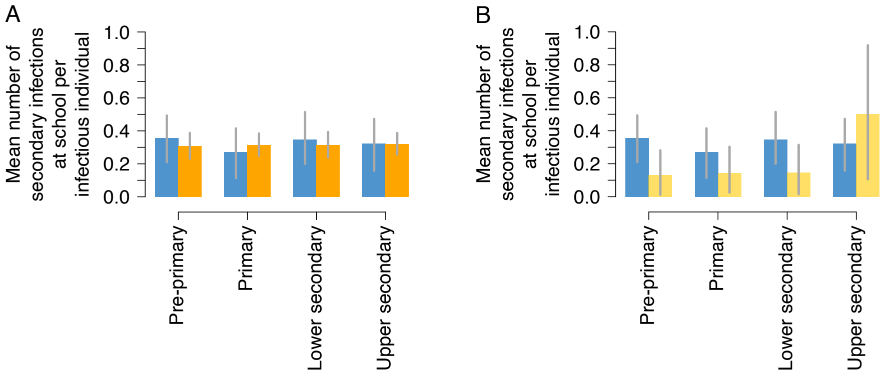

Figure S10. Mean number of secondary infections at school per infectious individual by educational level. A. Main model with homogeneous susceptibility by age. **B.** Alternative model calibration after assuming an age-heterogeneity in susceptibility [36]. Bars: mean estimates; vertical lines: 95%CrI.

References

[1] Manica M, Litvinova M, De Bellis A, et al. Estimation of the incubation period and generation time of SARS-CoV-2 Alpha and Delta variants from contact tracing data. *Epidemiol Infect* 2022;**151**:e5. https://doi.org/10.1017/S0950268822001947

[2] Molina Grané C, Mancuso P, Vicentini M, et al. SARS-CoV-2 transmission patterns in educational settings during the Alpha wave in Reggio-Emilia, Italy. *Epidemics* 2023;**44**:100712. https://doi.org/10.1016/j.epidem.2023.100712

[3] Istituto Superiore di Sanità. *Monitoraggio delle varianti del virus SARS-CoV-2 di interesse in sanità pubblica in Italia*. https://www.epicentro.iss.it/coronavirus/sars-cov-2-monitoraggio-varianti-indagini-rapide (11 June 2026, date last accessed).

[4] Djuric O, Larosa E, Cassinadri M, et al. Surveillance, contact tracing and characteristics of SARS-CoV-2 transmission in educational settings in Northern Italy, September 2020 to April 2021.
PLoS One 2022;**17(10)**:e0275667. https://doi.org/10.1371/journal.pone.0275667

[5] Merler S, Ajelli M. The role of population heterogeneity and human mobility in the spread of pandemic influenza. *Proc Biol Sci* 2009;**277(1681)**:557-65. https://doi.org/10.1098/rspb.2009.1605

[6] Merler S, Ajelli M, Pugliese A, et al. Determinants of the spatiotemporal dynamics of the 2009 H1N1 pandemic in Europe: implications for real-time modelling. *PLOS Comput Biol* 2011;**7(9)**:e1002205. https://doi.org/10.1371/journal.pcbi.1002205

[7] Fumanelli L, Ajelli M, Manfredi P, et al. Inferring the Structure of Social Contacts from Demographic Data in the Analysis of Infectious Diseases Spread. *PLOS Comput Biol* 2012;**8(9)**:e1002673. https://doi.org/10.1371/journal.pcbi.1002673

[8] Fumanelli L, Ajelli M, Merler S, et al. Model-based Comprehensive Analysis of School Closure Policies for Mitigating Influenza Epidemics and Pandemics. *PLOS Comput Biol* 2016;**12(1)**:e1004681. https://doi.org/10.1371/journal.pcbi.1004681

[9] Nasa's Socioeconomic Data and Applications Center (SEDAC). *Gridded Population of the World, Version 4 (GPWv4): Population count, Revision 11*. https://www.earthdata.nasa.gov/data/catalog/sedac-ciesin-sedac-gpwv4-popcount-r11-4.11 (11 June 2026, date last accessed).

[10] Statistical office of the European Commission (Eurostat). *Database*. https://ec.europa.eu/eurostat/data/database (11 June 2026, date last accessed).

[11] Istituto Italiano di Statistica. *Nidi e servizi integrativi per la prima infanzia – anno educativo 2019/2020*. https://www.istat.it/it/files/2021/11/REPORT_ASILI-NIDO-2019-2020.pdf (11 June 2026, date last accessed).

[12] Istituto Italiano di Statistica. *Istruzione e formazione – scuole*. https://esploradati.istat.it/databrowser/#/it/dw/categories/IT1,Z0820EDU,1.0/DCIS_SCUOLE (11 June 2026, date last accessed).

[13] UNESCO Institute for Statistics. *Other policy relevant indicators – Education*. https://databrowser.uis.unesco.org/browser/EDUCATION/UIS-EducationOPRI (11 June 2026, date last accessed).

[14] Education, Audiovisual and Culture Executive Agency, European Commission. *Key data on education in Europe 2009* https://ec.europa.eu/eurostat/web/products-statistical-books/-/978-92-9201-033-1 (11 June 2026, date last accessed).

[15] Openpolis. *Geojson Italy*. https://github.com/openpolis/geojson-italy (11 June 2026, date last accessed).

[16] Education, Audiovisual and Culture Executive Agency, European Commission. *Key data on education in Europe 2012*. https://eurydice.eacea.ec.europa.eu/publications/key-data-education-europe-2012 (11 June 2026, date last accessed).

[17] Organisation for Economic Co-operation and Development (OECD). *Education at a glance 2024 - Ratio of students to teaching staff by type of insititution*. https://www.oecd.org/en/publications/education-at-a-glance-2024_c00cad36-en.html (11 June 2026, date last accessed).

[18] Ufficio scolastico regionale per l'Emilia-Romagna. *Calendario scolastico a.s. 2020-21*. https://www.istruzioneer.gov.it/2020/07/22/calendario-scolastico-a-s-2020-21/ (11 June 2026, date last accessed).

[19] Gonzalez MC, Hidalgo CA, Barabasi AL Understanding individual human mobility patterns. *Nature* 2008;**453(7196)**:779–82. https://doi.org/10.1038/nature06958

[20] Merler S, Ajelli M, Fumanelli L, et al. Containing the accidental laboratory escape of potential pandemic influenza viruses. *BMC Med* 2013:**11**:252. https://doi.org/10.1186/1741-7015-11-252

[21] Poletti P, Tirani M, Cereda D, et al. Association of age with likelihood of developing symptoms and critical disease among close contacts exposed to patients with confirmed SARS-CoV-2 infection in Italy. *Jama Netw Open* 2021;**4(3)**:e211085. https://doi.org/10.1001/jamanetworkopen.2021.1085

[22] Marziano V, Guzzetta G, Menegale F, et al. Estimating SARS-CoV-2 infections and associated changes in COVID-19 severity and fatality. *Influenza Other Respir Viruses* 2023;**17(8)**:e13181. https://doi.org/10.1111/irv.13181

[23] Djuric O, Larosa E, Cassinadri M, et al. Effect of an enhanced public health contact tracing intervention on the secondary transmission of SARS-CoV-2 in educational settings: The four-way decomposition analysis. *Elife* 2024;**13**:e85802. https://doi.org/10.7554/eLife.85802

[24] Regione Emilia-Romagna. *Ordinanza del Presidente della Giunta regionale 6 aprile 2021, N. 43*. https://www.inapp.gov.it/wp-content/uploads/NORMATIVA/2021/Regionale/20210406_OrdinanzaPresidenteGiuntaRegionale_n43_ER.pdf (11 June 2026, date last accessed).

[25] Presidenza del Consiglio dei Ministri della Repubblica Italiana. *Decreto del Presidente del Consiglio dei Ministri del 3 novembre 2020*. https://www.gazzettaufficiale.it/eli/id/2020/11/04/20A06109/sg (11 June 2026, date last accessed).

[26] Presidenza del Consiglio dei Ministri della Repubblica Italiana. *Decreto del Presidente del Consiglio dei Ministri del 2 marzo 2021*. https://www.gazzettaufficiale.it/eli/id/2021/03/02/21A01331/sg (11 June 2026, date last accessed).

[27] Presidente della Repubblica Italiana. *Decreto-legge 1 aprile 2021, n.44*. https://www.gazzettaufficiale.it/eli/id/2021/04/01/21G00056/sg (11 June 2026, date last accessed).

[28] Toni T, Welch D, Strelkowa N, et al. Approximate Bayesian computation scheme for parameter inference and model selection in dynamical systems. *J R Soc Interface* 2008;**6(31)**:187–202. https://doi.org10.1098/rsif.2008.0172

[29] Guzzetta G, Marziano V, Mammone A, et al. The decline of the 2022 Italian mpox epidemic: Role of behavior changes and control strategies. *Nat Commun* 2024;**15(1)**:2283. https://doi.org/10.1038/s41467-024-46590-4

[30] Manica M, Guzzetta G, Riccardo F, et al. Impact of tiered restrictions on human activities and the epidemiology of the second wave of COVID-19 in Italy. *Nat Commun* 2021;**12(1)**:4570. https://doi.org/10.1038/s41467-021-24832-z

[31] Cereda D, Manica M, Tirani M, et al. The early phase of the COVID-19 epidemic in Lombardy, Italy. *Epidemics* 2021;**37**:100528. https://doi.org/10.1016/j.epidem.2021.100528

[32] Trentini F, Marziano V, Guzzetta G, et al. Pressure on the health-care system and intensive care utilization during the COVID-19 outbreak in the Lombardy region of Italy: a retrospective observational study in 43,538 hospitalized patients. *Am J Epidemiol* 2022;**191(1)**:137-46. https://doi.org/10.1093/aje/kwab252

[33] Peto T, UK COVID-19 Lateral Flow Oversight Team. COVID-19: Rapid antigen detection for SARS-CoV-2 by lateral flow assay: A national systematic evaluation of sensitivity and specificity for mass-testing. *EClinicalMedicine* 2021;**36**:100924. https://doi.org10.1016/j.eclinm.2021.100924

[34] Mistry DA, Wang JY, Moeser ME et al. A systematic review of the sensitivity and specificity of lateral flow devices in the detection of SARS-CoV-2. *BMC Infect Dis* 2021;**21(1)**:828. https://doi.org/10.1186/s12879-021-06528-3

[35] World Health Organization. *Antigen-detection in the diagnosis of SARS-CoV-2 infection. Interim guidance*. https://iris.who.int/bitstream/handle/10665/345948/WHO-2019-nCoV-Antigen-Detection-2021.1-eng.pdf (11 June 2026, date last accessed).

[36] Zhang J, Litvinova M, Liang Y, et al. Changes in contact patterns shape the dynamics of the novel coronavirus disease 2019 outbreak in China. *Science* 2020;**368(6498)**:1481–86. https://doi.org/10.1126/science.abb8001
